# Computer-based cognitive interventions for mild cognitive impairment and dementia in older adults: protocol for a systematic review of published studies and meta-analysis

**DOI:** 10.1186/s13643-019-1146-x

**Published:** 2019-09-06

**Authors:** Abdullah Al Mahmud, Reneta Slikboer, Jennifer Stargatt, Sunil Bhar

**Affiliations:** Swinburne University of Technology, Melbourne, Australia

**Keywords:** Dementia, Cognition, Impairment, Computer-based intervention, Technology, Stimulation, Therapy

## Abstract

**Background:**

A growing number of older adults experience mild cognitive impairment (MCI) and dementia. Recent technological advances allow for traditional cognitive interventions to be administered via computers and other devices. The aim of the proposed systematic review and meta-analyses is to determine the efficacy of computerised cognitive interventions for MCI and dementia in older adults.

**Methods:**

We will systematically search electronic databases and reference lists to identify randomised controlled trials. We will include studies that examine the use of computer-based cognitive interventions for adults aged over 60 with MCI or dementia. Primarily outcomes are global and domain-specific cognitive function. Secondary outcomes are attitudes (usability, understandability, acceptability of the intervention), mood and quality of life. Risk of bias will be assessed. Finally, the summary effect sizes will be reported.

**Discussion:**

This systematic review will summarise existing high-quality primary studies on computerised-cognitive interventions for MCI and dementia. Results from this review will provide the basis for future research in developing computer-based interventions for this population.

**Systematic review registration:**

PROSPERO CRD42016050236

**Electronic supplementary material:**

The online version of this article (10.1186/s13643-019-1146-x) contains supplementary material, which is available to authorized users.

## Background

Globally, the number of older persons—aged 60 and over—is growing faster than any other age group [1]. Therefore, the prevalence of later-life cognitive disorders can be expected to increase. Dementia, or major neurocognitive disorder (DSM-5), is the significant impairment of cognitive performance in one or more cognitive domains (e.g. complex attention, learning and memory, executive function, language), ultimately resulting in functional incapacity and death [2]. The most common cause of dementia is Alzheimer’s disease, where it affects 6–9% of adults aged over 60 worldwide [3]. Mild cognitive impairment (MCI) involves cognitive decline that is greater than that which occurs in normal ageing, with some limitations to daily function (*Statistical Manual of Mental Disorders* (fifth ed.; *DSM-5*). It is estimated that MCI affects up to 42% of older adults and precedes the onset of dementia [4].

Studies have indicated that computer-based interventions may be beneficial for improving or maintaining cognitive function to slow the trajectory of cognitive decline, for both those with MCI and dementia [5–10]. In recent years, advances in computer technology have allowed such interventions to be administered using personal computers, laptops, tablets and other mobile devices, in an increasingly accessible, individualised and cost-effective manner. The usefulness of computer-based interventions has also been demonstrated in meta-analyses [11, 12]. However, these meta-analyses have been associated with methodological limitations. For example, Garcia-Casal et al. [11] used a fixed-effects statistical model to calculate summary effect sizes. Such a model assumes that all differences between studies can be accounted for by sampling error [13]. Hence, this study may have unintentionally inflated effect sizes. To correct for such inflation, Hill et al. [12] used a random-effects statistical model [13] but failed to discriminate between active and passive control groups in their statistical analysis. Similarly, Sitzer et al. [14] included mixed treatment types (e.g. computerised training with exercise); therefore, were not able to distinguish the effects of computerised training from those associated with adjunct interventions.

### Aim

We aim to conduct a systematic review and meta-analysis that uses a random effects model and that compares the effects of computerised training that is not mixed with other interventions, with three types of control groups—placebo, active and passive. The review will consider the effects of such training for individuals diagnosed with dementia and MCI. The purpose of this systematic review protocol is to transparently present the method we will undertake in order to conduct the systematic review, such that it could adequately be replicated. This method includes information regarding eligibility criteria, information sources and search strategy to be used, and the process of data extraction, synthesis and analysis.

## Method

### Protocol and registration

This protocol was developed in adherence with the Preferred Reporting Items for Systematic Review and Meta-Analysis Protocols (PRISMA-P) guidelines [15], see Additional file 1 for the PRISMA-P checklist. The review will also adhere to the guidelines specified by the Preferred Reporting Items for Systematic Reviews and Meta-Analyses (PRISMA) [16]. This review has been registered with the International Prospective Register of Systematic Reviews (PROSPERO); CRD42016050236.

### Eligibility criteria

#### Study design

We will include published randomised controlled trials. Other study designs will be excluded to minimise the influence of selection and reporting bias on summary effect sizes [17].

#### Participants

The target population is older adults (> 60 years old) who have a diagnosis of dementia or MCI, confirmed by a trained practitioner (e.g. medical practitioner). Studies that include participants below the age of 60 will be included if the mean age of participants is 60 or over [18].

#### Intervention

Included interventions will be computer-based, i.e. delivered on personal computers, laptops and other devices such as tablets and mobile phones, and may be categorised as cognitive stimulation, cognitive recreation, cognitive rehabilitation, cognitive training [11], or cognitive remediation. The intervention must be designed to improve cognitive function and may be of any length and intensity. Dual interventions, e.g. combined cognitive training and medication will be excluded [18].

#### Comparator groups

Any type of control group may be included in the review, including placebo, active and passive conditions. Examples of such groups are care-as-usual, non-computerised cognitive training, pharmacological and waitlist conditions. Participants on their usual treatment medication will not be excluded [18].

#### Outcomes

The primary outcomes are global cognitive functioning (measured by tests such as the Mini-Mental State Examination) and domain-specific cognitive function (measured by tests such as Hopkins Verbal Learning Test-Revised for memory and the Delis-Kaplin Executive Function System for executive function). Secondary outcome measures will be participant attitudes toward the intervention (e.g. usability, understandability and acceptability), mood and quality of life.

### Search methods for identification of eligible studies

The electronic search strategy was guided by the Cochrane Handbook of Systematic Reviews of Interventions [18]. We will search The Cochrane Central Register of Controlled Trials (CENTRAL) [19], Scopus (which includes most of Embase), CINAHL (via EBSCOhost [20]), MEDLINE (via PubMed) and ALOIS. Standard filters for identifying RCTs will be applied to CINAHL and MEDLINE.

Search terms were guided by previous meta-analyses [11, 12]. Search terms were chosen to describe the concepts of the health condition, the intervention and the study design included in this review. The references of studies meeting the inclusion criteria and meta-analyses found on the topic of interest will be screened by hand. Preliminarily searches have been conducted to ensure the scope of this review is viable, see **Additional file** **2** for results of the preliminarily search. An example preliminary search of Cochrane Central revealed 437 records based on the following terms: geriatric OR old* OR elder* OR “late* life” OR senior AND “mild cognitive impairment” OR dementia AND “cognitive training” OR “cognitive rehabilitation” OR “cognitive remediation” OR “brain training” OR “computer training” OR “brain games” OR “brain exercise” OR “cognitive therapy” OR “cognitive treatment” OR “cognitive interventions”.

### Data collection and analysis

#### Selection of studies

Studies must be in English and can be from any country of origin to be included. Two authors will screen the search results at the title, abstract and full-text level [18]. We will use online screening and extraction software Covidence to conduct all stages of screening [21]. Discrepancies between the reviewers’ decisions will be resolved by reaching consensus or by obtaining the opinion of a third reviewer. Reasons for excluding studies at the full-text stage will be recorded. A flow chart illustrating the search and selection process of the review will be included in the final review and inter-rater reliability statistics will be reported.

#### Data extraction and management

We will extract pre-defined data from studies chosen for inclusion in the review using a paper-based data extraction form.

1. Publication details: authors, title, journal, year, geographical location in which the study was conducted and funding source.
2. Study design: type of study (e.g. RCT)
3. Participant details: sample size, demographic information (e.g. age and gender of participants), condition (e.g. MCI or dementia), participant drop out/completion rates.
4. Intervention details: the Tidier Checklist [22] will be used to guide the extraction of the intervention details, with particular attention to technology and software used, the aim of the intervention, dosage and timing of the intervention, information regarding the comparator group(s).
5. Outcome details: data for primary and secondary outcomes.

Two authors will extract data from the included studies; disagreements will be resolved by a third author. We will contact study authors to obtain further information where necessary. The data will then be exported into the Review Manager (RevMan) program for further analysis [23]. The extracted data will be made available to the public.

#### Assessment of risk of bias in included studies

To assess the risk of bias of individual studies, two reviewers will use the risk of bias assessment procedure in RevMan independently. Disagreement will be resolved by consensus or a third reviewer and inter-rater reliability statistics will be conducted.

#### Measures of treatment efficacy

It is expected that different measures will be used to assess treatment efficacy; therefore, we will use the standardised mean difference. This is calculated by dividing a study’s mean difference by that study’s standard deviation [13]. Separate analyses will be conducted for different types of dementia and level of severity (MCI and dementia) using a random effects model.

#### Dealing with missing data

We will contact authors for missing data and clarity of primary studies if required, such inclusions will be reported in the review.

#### Data synthesis

Studies will be meta-analysed using the RevMan software [23]. We will meta-analyse studies that report our outcomes of interest. The meta-analyses will be conducted using an inverse-variance, random-effects model. We will calculate 95% confidence intervals and two-sided *p* values for each outcome. We will include forest plots to display the results of the meta-analyses. Where it is not appropriate to include studies in a meta-analysis, we will provide a narrative summary.

#### Assessment of heterogeneity

Statistical heterogeneity will be examined using the chi-squared test and the *I*^2^ statistic, and it will be examined for all studies included. A sensitivity analysis will be conduct between fixed-effects and random-effects models; relevant differences will be reported.

#### Assessment of reporting biases

We will examine funnel plots of the included studies to evaluate potential publication bias.

#### Assessment of the quality of the evidence

We will use the GRADE approach (Grading of Recommendation, Assessment, Development and Evaluation), as described in the Cochrane Handbook for Systematic Reviews and Interventions to evaluate the quality of the body of evidence [18]. Following the GRADE guidelines, rating the quality of the body of evidence for each outcome involves consideration of limitations in study design and implementation, the directness of evidence, heterogeneity/inconsistency of results, the precision of results demonstrated by confidence intervals and the probability of publication bias. A summary of evidence based on these guidelines will be provided for all meta-analysed outcomes in the review.

## Discussion

The rapidly growing number of older adults worldwide is likely to increase in the prevalence of neurocognitive disorders such as MCI and dementia. Computer-based cognitive interventions may be effective for improving the lives of older adults with MCI and dementia and may be favoured over traditional interventions for their accessibility and cost-effectiveness.

Observing the methods detailed in this protocol, the review will present up-to-date evidence regarding the effect of computer-based cognitive interventions for older adults with MCI and dementia. The systematic review will be reported according to the PRISMA guidelines [16] and submitted for publication to an appropriate peer-reviewed journal. The findings of the systematic review will serve as a basis for further research regarding the development of computer applications for MCI and dementia.

## Limitations

There are two clear limitations to the current review protocol. The review will be restricted to published studies; publication bias is expected to be detected by funnel plots. Secondly, only studies in the English language will be included, introducing language bias.

## Additional files

Additional file 1: PRISMA-P checklist. (PDF 235 kb)

Additional file 2: Detailed search method (PDF 180 kb)

## Data Availability

Not applicable.

## Abbreviations

DSM-5: Diagnostic and Statistical Manual of Mental Disorders, Fifth Edition
MCI: Mild cognitive impairment
