# Supplementary material for: Computer-based cognitive interventions for mild cognitive impairment and dementia in older adults: protocol for a systematic review of published studies and meta-analysis
Source: Syst Rev. 2019 Sep 6;8:231. doi: 10.1186/s13643-019-1146-x (PMC6729043; doi:10.1186/s13643-019-1146-x)
Supplement: Supplementary file 2 — Detailed search method (PDF 180 kb) [file 13643_2019_1146_MOESM2_ESM.pdf]

Detailed search method

May 2019

Cochrane Controlled Register of Trials Central (CENTRAL)

437

geriatric OR old* OR elder* OR “late* life” OR senior AND “mild cognitive impairment” OR dementia AND “cognitive training” OR “cognitive rehabilitation” OR “cognitive remediation” OR “brain training” OR “computer training” OR “brain games” OR “brain exercise” OR “cognitive therapy” OR “cognitive treatment” OR “cognitive interventions”

Scopus

417

geriatric OR old* OR elder* OR “late* life” OR senior AND “mild cognitive impairment” OR dementia AND “cognitive training” OR “cognitive rehabilitation” OR “cognitive remediation” OR “brain training” OR “computer training” OR “brain games” OR “brain exercise” OR “cognitive therapy” OR “cognitive treatment” OR “cognitive interventions” AND “random* control* trial” OR “clinical trial”

CINAHL Complete (via EBSCOhost, CINAHL filter for RCTs)

233

geriatric OR old* OR elder* OR “late* life” OR senior AND “mild cognitive impairment” OR dementia AND “cognitive training” OR “cognitive rehabilitation” OR “cognitive remediation” OR “brain training” OR “computer training” OR “brain games” OR “brain exercise” OR “cognitive therapy” OR “cognitive treatment” OR “cognitive interventions” AND MH randomized controlled trials OR MH single- blind studies OR MH random assignment OR MH pretest- posttest design OR MH cluster sample OR TI (randomised OR randomized) OR AB random* OR TI trial OR MH ((sample size) AND AB (assigned OR allocated OR control)) OR MH placebos OR PT randomized controlled trial OR AB cluster W3 RCT OR human

Pubmed

227

(((geriatric OR old* OR elder* OR “late* life” OR senior AND “mild cognitive impairment” OR dementia AND “cognitive training” OR “cognitive rehabilitation” OR “cognitive remediation” OR “brain training” OR “computer training” OR “brain games” OR “brain exercise” OR “cognitive therapy” OR “cognitive treatment” OR “cognitive interventions”))) AND (((((((randomized controlled trial[Publication Type]) OR controlled clinical trial[Publication

Type]) OR randomized[Title/Abstract]) OR placebo[Title/Abstract]) OR clinical trials[MeSH Major Topic]) AND randomly[Title/Abstract]) AND trial[Title])

ALOIS

Dementia – 543

MCI - 94

No search terms were entered into the search box as terms defining our aged bracket lead to unsatisfacorty results. Rather, search results were limited by drop down menus. Study type – Intervention study; Current Status – Completed; Intervention Type – Non-pharmacological; Study Design – RCT; Study Aim – Treatment Dementia/Treatment MCI; Study Design (Diagnostic) – any.
